# Control of infection by LC3-associated phagocytosis, CASM, and detection of raised vacuolar pH by the V-ATPase-ATG16L1 axis

**DOI:** 10.1126/sciadv.abn3298

**Published:** 2022-10-26

**Authors:** Yingxue Wang, Maria Ramos, Matthew Jefferson, Weijiao Zhang, Naiara Beraza, Simon Carding, Penny P. Powell, James P. Stewart, Ulrike Mayer, Thomas Wileman

**Affiliations:** ^1^ Norwich Medical School, University of East Anglia, Norwich, UK.; ^2^ Quadram Institute Bioscience, Norwich, UK.; ^3^ Department of Infection Biology, University of Liverpool, Liverpool, UK.; ^4^ School of Biological Sciences, University of East Anglia, Norwich, UK.

## Abstract

The delivery of pathogens to lysosomes for degradation provides an important defense against infection. Degradation is enhanced when LC3 is conjugated to endosomes and phagosomes containing pathogens to facilitate fusion with lysosomes. In phagocytic cells, TLR signaling and Rubicon activate LC3-associated phagocytosis (LAP) where stabilization of the NADPH oxidase leads to sustained ROS production and raised vacuolar pH. Raised pH triggers the assembly of the vacuolar ATPase on the vacuole membrane where it binds ATG16L1 to recruit the core LC3 conjugation complex (ATG16L1:ATG5-12). This V-ATPase-ATG16L1 axis is also activated in nonphagocytic cells to conjugate LC3 to endosomes containing extracellular microbes. Pathogens provide additional signals for recruitment of LC3 when they raise vacuolar pH with pore-forming toxins and proteins, phospholipases, or specialized secretion systems. Many microbes secrete virulence factors to inhibit ROS production and/or the V-ATPase-ATG16L1 axis to slow LC3 recruitment and avoid degradation in lysosomes.

## Teaser

Conjugation of LC3 to vacuoles containing pathogens provides alternative ways for autophagy proteins to control infection.

## CANONICAL AUTOPHAGY AND ALTERNATIVE PATHWAYS OF LC3 CONJUGATION

**Fig. 1.**
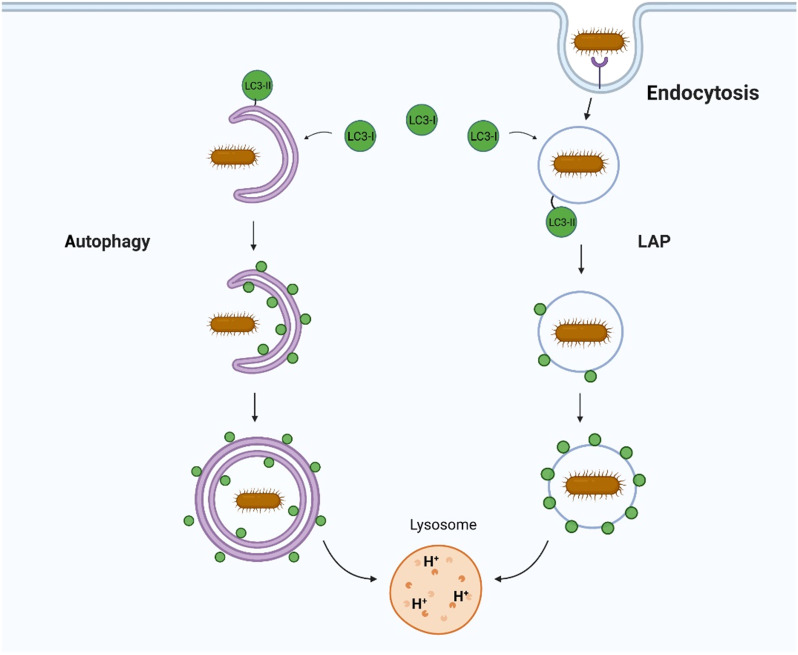
Autophagy and LAP provide different ways to control infection. Canonical autophagy conjugates LC3 to double-membrane autophagosomes that engulf pathogens in the cytosol. LAP conjugates LC3 to endosomes and phagosomes containing extracellular pathogens as they enter cells. In both cases, conjugation of LC3 to membranes containing pathogens facilitates fusion with lysosomes, leading to pathogen degradation.

Autophagy provides a powerful means of removing pathogens from cells (Fig. 1) by degrading them in lysosomes. The best studied pathway is called “canonical” autophagy that uses a series of autophagy (ATG) proteins to generate autophagosomes that capture pathogens in the cytoplasm (*1*). Pathogens are killed and degraded when autophagosomes fuse with lysosomes, allowing microbial components to be exposed to innate and acquired immune systems. Many pathogens enter cells by endocytosis or phagocytosis, and during this early phase of infection, they are protected from canonical autophagy by the endosome/phagosome membrane. Viruses use this opportunity to release genomes into the cell, and several bacteria and parasites modify the phagosome/endosome membranes to provide vacuoles specialized for replication. In many cases, this gap in microbial defense has been filled by alternative pathways that use a subset of ATG proteins to target endosomes and phagosomes containing microbes. A key feature of both pathways is the conjugation of autophagy protein LC3/ATG8 (LC3) to the membranes surrounding the pathogen to facilitate fusion with lysosomes.

## LAP, LANDO, AND CASM

The terminology used for alternative pathways of LC3 conjugation can be rationalized by considering the origins of the material being delivered to the lysosome (Fig. 1). In the context of infection, classical canonical autophagy delivers pathogens directly from the cytosol to lysosomes and involves conjugation of LC3 to double-membrane autophagosomes. In contrast, the alternative pathways result in conjugation of LC3 to single-membrane endosomes and/or phagosomes, allowing pathogens to be delivered to lysosomes before they reach the cytosol. Alternative LC3 conjugation pathways were discovered during early studies that noticed the recruitment of LC3 from the cytosol to phagosomes generated in macrophages ingesting killed yeast, *Escherichia coli* bacteria, or incubated with ligands for Toll-like receptors (TLRs) (*2*, *3*). LC3 recruitment was dependent on proteins essential for autophagy such as ATG5 and ATG7, but unexpectedly, LC3 was recruited to single-membrane phagosomes rather than double-membrane autophagosomes. This alternative pathway was called LC3-associated phagocytosis (LAP) to indicate a pathway operating in phagocytic cells to eliminate pathogens and apoptotic cells. Similar studies showed that recruitment of LC3 to phagosomes in phagocytic cells and to vacuoles containing *Salmonella* in epithelial cells was dependent on NADPH (reduced form of nicotinamide adenine dinucleotide phosphate) oxidase (*4*). This showed that recruitment of LC3 to endocytic membranes was not unique to phagocytic cells and could take place in many different cell types. LC3 recruitment could also be induced by a wide range of stimuli. Examples now include perturbation of endolysosome membranes when the osmotic balance within endocytic vesicles is perturbed by lysosomotropic drugs, by inhibitors of the vacuolar adenosine triphosphatase (V-ATPase), by stimulation of lysosome Ca^2+^ channel TRPML1, or during uptake of transfection reagents and apoptotic cells or following uptake of exocytic zymogen granules by pancreatic acinar cells (*5*–*10*). For this reason, an umbrella term CASM has been proposed to describe the conjugation of ATG8 to endolysosomal single membranes (*11*). LC3 recruitment also influences endocytic trafficking and has been called LANDO to describe LC3-associated endocytosis. LANDO is implicated in the clearance of β-amyloid and pathogenesis of Alzheimer’s disease, and during endocytosis and signaling of cytokine receptors (*12*–*14*).

**Fig. 2.**
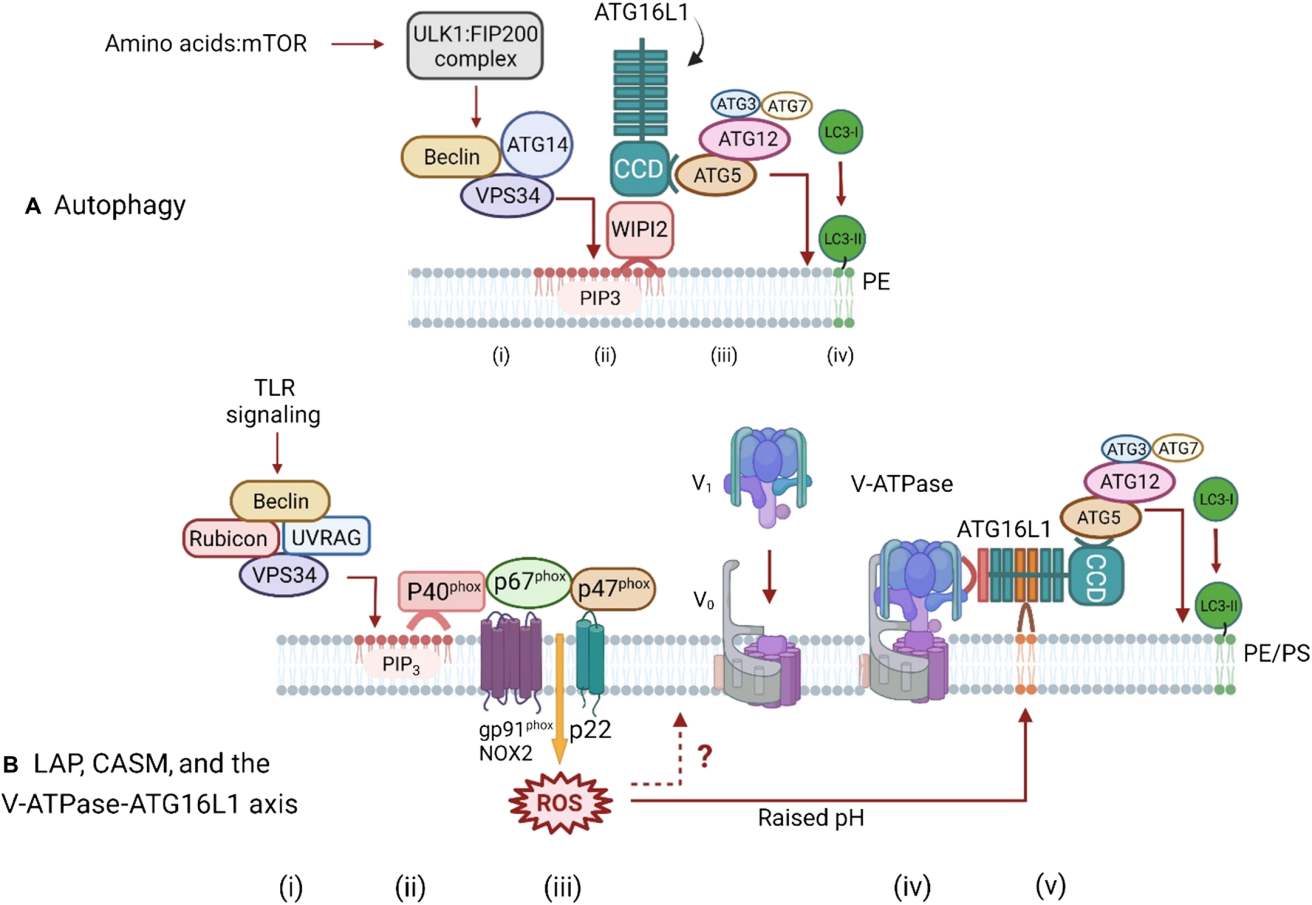
Pathways for ATG16L1-mediated conjugation of LC3 to membranes during autophagy and LAP/CASM conjugation via the V-ATPase-ATG16L1 axis. (**A**) Autophagy. Autophagy is activated in response to a fall in amino acids, which leads to inhibition of mammalian target of rapamycin (mTOR) and activation of the ULK1/FIP200 initiation complex (i) and downstream activation of the PI3K complex containing Beclin1, ATG14, and VPS34. The PI3K activity of VPS34 generates PIP_3_ in autophagosome membranes, which provide a platform for binding of WIPI2 (ii). WIPI2 binds to the coiled coil domain (CCD) of ATG16L1, leading to recruitment of the LC3 conjugation complex (ATG5-ATG12, ATG3, and ATG7). The conjugation reaction results in conversion of LC3I to LC3II and covalent binding of LC3 to PE in the autophagosome membrane (iii and iv). (**B**) LAP/CASM and the V-ATPase-ATG16L1 axis. LC3 conjugation in phagocytic cells is activated by TLR signaling through a complex containing Rubicon, Beclin1, UVRAG, VPS15, and VPS34 (i). TLR signaling activates VPS34 within the complex leading to generation of PIP_3_ in phagosome membranes (ii) to generate a binding site for p40^phox^ that stabilizes the NADPH complex (NOX2, p47^phox^, p40^phox^, and p67^phox^). At the same time, binding of Rubicon to p22phox increases production of reactive oxygen species (ROS). Generation of ROS increases the pH in the lumen of the phagosome (iii) stimulating assembly of the V_o_ V_1_ subunits of V_o_-V_1_ (iv). It is also possible that ROS can act directly on V-ATPase (?). V-ATPase binds to the WD domain of ATG16L1, leading to recruitment of the LC3 conjugation complex (ATG5-ATG12, ATG3, and ATG7) to the phagosome and conjugation of LC3 to phosphatidylserine (PS) and phosphatidylethanolamine (PE) in the phagosome membrane (v). A similar ROS-dependent pathway involving assembly of V-ATPase operates in nonphagocytic cells, but the precise components of the NADPH oxidase complex are unclear.

The main differences between canonical autophagy and alternative LAP, LANDO, and CASM pathways are summarized in Fig. 2. In both cases, conjugation of LC3 to phospholipids in lipid bilayers involves the core LC3/ATG8 lipidation complex composed of ATG5-ATG12, ATG16L1, and ATG3/ATG7, but they differ in upstream signaling. During canonical autophagy (Fig. 2A), activation of the ULK1 complex (ULK1, FIP200, ATG13, and ATG101) in response to a fall in amino acids leads to activation of the “PI3K complex” containing VPS15, VPS34, Beclin1, and ATG14 (*15*, *16*). The phosphatidylinositol 3-kinase (PI3K) activity of VPS34 generates phosphatidylinositol 3,4,5-trisphosphate (PIP_3_) in lipid bilayers to generate a binding site for WIPI2, which, in turn, binds the ATG16L1:ATG5-ATG12 complex to initiate conjugation of LC3 to phosphatidylethanolamine (PE) (*17*).

In contrast, LAP is activated by TLR signaling and mediated by a complex of proteins containing Rubicon, Beclin1, UVRAG, VPS15, and VPS34 (Fig. 2B). TLR signaling induces binding of Rubicon to the p22phox subunit of the NADPH oxidase complex to increase production of reactive oxygen species (ROS) within the phagosome. At the same time, generation of PIP_3_ by VPS34 generates a binding site for the N-terminal phox-homology (PX) domain in p40^phox^ to stabilize the NADPH complex. Sustained production of ROS increases the pH within the phagosome, and this is thought to provide a signal for recruitment of the ATG16L1:ATG5-ATG12 complex to the phagosome to initiate conjugation of LC3 to the amino residues of PE and phosphatidylserine (PS), which is abundant in phagosome membranes (*11*, *18*–*20*). ATG4 is a cysteine protease able to cleave LC3 at the C-terminal glycine-120 used for attachment to PE or PS to recycle LC3 back to the cytosol. Production of ROS during LAP inhibits ATG4 to slow removal of LC3 from the phagosome membrane and increase processing and presentation of antigens on class II major histocompatibility complex (MHC) (*21*).

## ALTERNATIVE PATHWAYS OF LC3 LIPIDATION OFTEN REQUIRE THE WD DOMAIN OF ATG16L1

The canonical autophagy and alternative pathways use different domains of ATG16L1 to recruit the ATG16L1:ATG5-ATG12 complex to membranes (Fig. 2). Canonical autophagy requires the N-terminal coiled coil domain (CCD) of ATG16L1, which binds ATG5-ATG12 and WIPI2. This directs ATG16L1:ATG5-ATG12 to sites enriched for PIP_3_ generated in autophagosome membranes by VPS34. The C terminus of ATG16L1 contains a WD repeat domain that is not required for autophagy but is required for recruitment of ATG16L1:ATG5-ATG12 during LAP/CASM (*22*, *23*). The WD domain contains a lysine residue at position 490 that is crucial for lipidation of LC3 on endolysosome compartments (*22*). The WD domain also has several amino acids that can bind phospholipids “in vitro” and are required for binding ATG16L1 to perturbed endosome membranes (*24*).

## THE V-ATPase-ATG16L1 AXIS AND VAIL

Recent studies show that recruitment of the ATG16L1:ATG5-ATG12 complex onto endolysosome compartments is triggered by the assembly of V-ATPase. V-ATPase is anchored in endolysosome compartments by the integral membrane subunits of the V_o_ domain that make a pore to transport protons into the lumen of the vacuole, while the ATPase activity required for proton transport is provided by the V_1_ domain recruited from the cytosol. Recruitment of the V_1_ domain is triggered by a rise in vacuolar pH (Fig. 2B). Assembly of V-ATPase provides a binding site for ATG16L1, which recruits the LC3 conjugation machinery (ATG16L1:ATG5-ATG12) onto vacuoles. This pathway has been called the V-ATPase-ATG16L1 axis and/or VAIL for V-ATPase-ATG16L1–induced LC3B lipidation and was first identified during a study of recruitment of LC3 to vacuoles during *Salmonella* infection (*25*, *26*). Recruitment of LC3 during *Salmonella* infection required the core LC3 conjugation machinery (ATG3, ATG5:ATG12, ATG7, and ATG16L1) but was independent of FIP200, suggesting a pathway similar to CASM or LAP. This was confirmed by a CRISPR-Cas9 screen showing that recruitment of LC3 to the vacuole required V-ATPase, and that this involved binding of ATG16L1 to V-ATPase on the vacuole membrane (*26*). Furthermore, binding to V-ATPase was dependent on the ATG16L1 WD domain.

Subsequent work has demonstrated the importance of the interaction between the WD domain of ATG16L1 and V-ATPase for other conjugation pathways described under the CASM umbrella (*20*). Importantly, this has provided a link between the V-ATPase-ATG16L1 axis and ROS production by the NADPH oxidase (Fig. 2B) where the raised vacuolar pH induced by ROS drives assembly of the V_o_-V_1_ complex and subsequent recruitment of ATG16L1 (*20*, *27*). ATG16L1 can also be recruited to endocytic compartments by overexpression of TMEM59, a type 1 membrane glycoprotein found in late endosomes and lysosomes (*28*). The cytoplasmic domain of TMEM59 contains a short 19–amino acid domain that binds directly to the WD domain of ATG16L1 and is required for recruitment of LC3 to endosomes containing *Staphylococcus aureus*.

## THE V-ATPase-ATG16L1 AXIS IS ACTIVATED DURING STING SIGNALING

**Fig. 3.**
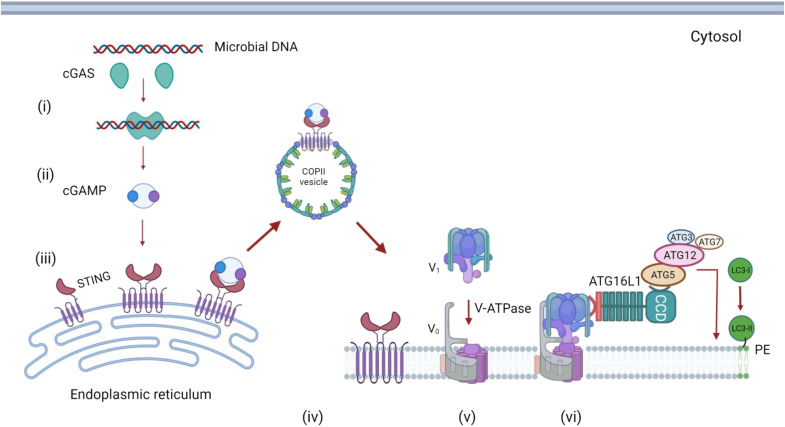
Recruitment of LC3 to membranes during STING signaling. Microbial DNA in the cytoplasm activates cGAS (**i**). The cyclic GM-AMP synthase activity of cGAS generates cyclic GMP-AMP (cGAMP) (**ii**). Binding of cGAMP to STING releases STING from the ER, allowing transport in COPII-coated vesicles to Golgi membranes (**iii**). STING facilitates assembly of the V_o_-V_1_ subunits of V-ATPase (**iv**). V-ATPase binds to the WD domain of ATG16L1, leading to recruitment of the LC3 conjugation complex (ATG5-ATG12, ATG3, and ATG7) and conjugation of LC3 to PE in the vesicle membrane. The precise identity of the vesicle membranes remains unclear, but they could be derived from the Golgi or endosomes.

Infection by DNA viruses or bacteria can introduce DNA into the cytosol where it is recognized by a DNA sensor called cGAS [cyclic guanosine monophosphate (GMP)–adenosine monophosphate (AMP) synthase], which uses GMP and AMP to generate cyclic dinucleotide GMP-AMP (cGAMP) (*29*). cGAMP acts as second messenger that activates STING to induce type 1 interferon (IFN) signaling and the formation of LC3 puncta (Fig. 3) (*25*, *30*, *31*). Activation of STING by cGAMP increases clearance of herpes simplex virus 1 (HSV1) and pseudotyped HIV from cells independently of IFN, suggesting a role for STING-mediated LC3 lipidation in controlling infection. Activation of STING by cGAMP releases STING from the endoplasmic reticulum (ER), leading to COPII-dependent movement into Golgi membranes and from there to perinuclear vesicles close to the Golgi apparatus. The vesicles have a single membrane and recruit the ATG5-ATG12:ATG16L1 complex to conjugate LC3 to PE independently of ULK1/2:FIP200 complex, suggesting recruitment through CASM/LAP (*25*, *30*).

Interestingly, recent work shows that, in common with CASM, activation of STING signaling leads to recruitment of the V_1_ complex of V-ATPase from the cytosol to the perinuclear vesicles containing LC3 by a pathway dependent on the WD domain of ATG16L1 (*25*). Precisely how recruitment of LC3 to vesicles by STING controls infection has not been resolved (*20*, *25*). The perinuclear vacuoles can capture DNA from the cytosol and could therefore engulf microbes in the cytoplasm; alternatively, it is possible that the perinuclear vesicles are derived from the endolysosome system, allowing capture of viruses entering cells and their delivery to lysosomes (*30*). Interestingly, STING-mediated conjugation of LC3 to membranes can be demonstrated in *Nematostella vectensis*, a small sea anemone that evolved 500 million years ago (*30*). It is possible that the ability of STING to drive LC3 conjugation to membranes is very ancient and evolved before the emergence of type 1 IFN pathways in vertebrates.

## THE SORTING NEXIN 5–ATG14 AXIS AND VIRAL INFECTION

**Fig. 4.**
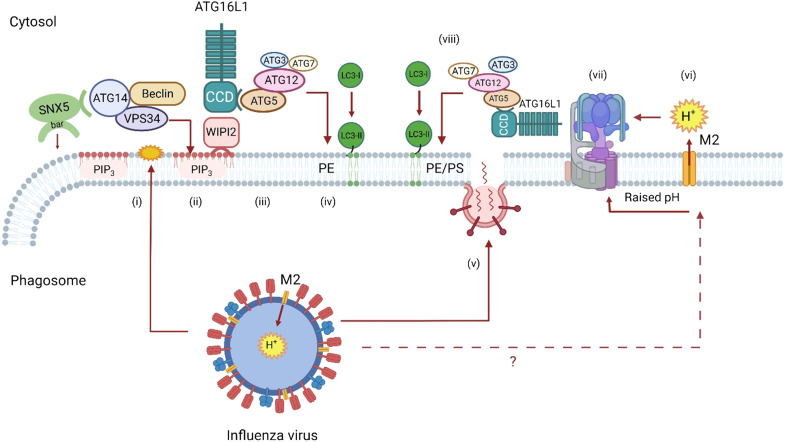
The SNX5-ATG14 axis and viral infection. The diagram uses influenza virus to illustrate responses to a broad range of viruses. Uptake of viruses into endosomes generates a signal (**i**) that recruits SNX5. SNX5 binds to ATG14 within the PI3K complex (VPS15, VPS34, Beclin1, and ATG14). VPS34 generates PIP_3_ in endosome membranes (**ii**), providing a binding site for WIPI2. The C-terminal BAR domain of SNX5 may sense (or generate) curvature in endosome membranes to enhance recruitment of ATG14 and generation of PIP_3_. WIPI2 binds to the CCD of ATG16L1, leading to recruitment of the LC3 conjugation complex (ATG5-ATG12, ATG3, and ATG7) (**iii**) and covalent binding of LC3 to PE and/or PS in the endosome membrane (**iv**). The V-ATPase-ATG16L1 axis may be activated when viruses generate pores in endosomes to facilitate delivery of genomes into the cytosol. This may involve fusion of capsids or envelopes with the endosome membrane (**v**) and/or synthesis of pore-forming proteins (viroporins). In the diagram, the M2 proton channel of influenza virus raises the pH of endosomes when inserted into the endosome membrane (**vi**). Raised pH leads to assembly of V-ATPase (**vii**) and recruitment of ATG16L1 and conjugation of LC3 to PE and/or PS (**viii**).

Infection of cells with viruses invariably leads to formation of LC3 puncta, indicating recruitment of LC3 to membrane compartments. Recent work using a genome-wide small interfering RNA (siRNA) screen shows that production of LC3 puncta in response to a broad range of viruses is reduced following silencing of sorting nexin 5 (SNX5) (*32*). Loss of SNX5 increased virus replication in tissue culture and increased susceptibility of neonatal *Snx5^−/−^* mice to lethal infection by several viruses. Interestingly, silencing of *Snx5* did not affect induction of canonical autophagy induced by starvation or following inhibition of mammalian target of rapamycin (mTOR). Furthermore, the recruitment of LC3 to vacuoles containing bacteria such as group A *Streptococcus*, or latex beads, or to membranes exposed to osmotic stress was independent of SNX5. Together, the results suggested that SNX5-dependent recruitment of LC3 to membranes is separate from LAP/CASM and represents a previously unknown pathway specialized to use LC3 conjugation to single-membrane vesicles (endosomes) as a defense against virus infection (Fig. 4).

Mechanistically, SNX5 binds endosomes through an N-terminal PX domain that recognizes PIP_3_ and a C-terminal concave BAR domain that binds to and/or induces curved membranes. Pull-down experiments show that SNX5 binds to ATG14/Barkor, an autophagy protein that forms part of the PI3K complex (VPS15, VPS34, Beclin1, and ATG14) required for canonical autophagy. Experiments using artificial lipids resembling endosomes showed that SNX5 increases generation of PIP_3_ by the PI3K complex containing ATG14, and this may involve the use of the rigid SNX5 BAR domain to increase membrane curvature, possibly providing a binding site for ATG14. The local production of PIP_3_ would then provide a binding site for WIPI2 and recruit the ATG16L1:ATG5-ATG12 LC3 conjugation machinery to endosome (Fig. 4). How viruses signal assembly of the SNX5:ATG14:PI3K complex on the cytosolic face of the endosome remains to be resolved. One possibility is that SNX5 binds to the cytoplasmic tails of transmembrane proteins that are used as receptors, or otherwise rearranged, during virus entry. If so, this would fit with the way that SNX5 regulates receptor trafficking during endocytosis.

## THE CASM/LAP TOOLBOX

Figure 1 shows that LAP and CASM pathways attach LC3 to single-membrane endosomes and phagosomes, while canonical autophagy attaches LC3 to double-membrane autophagosomes. This difference in membrane topology, and the ATG proteins required for conjugation of LC3 (Fig. 2), has proved very valuable in unraveling the roles played by different autophagy pathways during infection. Studies aimed at identifying LAP and CASM, for example, can use electron microscopy to demonstrate recruitment of LC3 to vesicles with a single membrane. LC3 recruitment should be independent of the unique components of the ULK1 initiation complex (ULK1, FIP200, and ATG13) required for autophagy, but require Rubicon or UVRAG, and this can be tested by gene silencing or isolation of cells [mouse embryonic fibroblasts (MEFs) and bone marrow–derived macrophages (BMDMs)] from knockout mice. Canonical autophagy and LAP/CASM can be further distinguished by establishing the requirement of the WD domain of ATG16L1 and the V-ATPase-ATG16L axis, and ROS. This can be achieved by expression of the SopF virulence factor from *Salmonella*, which blocks binding of the WD domain of ATG16L1 to V-ATPase, and drugs that promote (saliphenylhalamide) or inhibit (bafilomycin) assembly of the V_o_-V_1_ complex, and using diphenyleneiodonium (DPI) to inhibit NADPH oxidase (*20*, *26*).

## “IN VIVO” STUDIES

Thus far, two mouse models have been developed to study the consequences of LAP/CASM on pathogen survival in vivo. Mice lacking the WD and linker domains of ATG16L1 (ΔWD) are defective in LAP and CASM but preserve canonical autophagy through expression of the CCD of ATG16L1 that binds to WIPI2 (*23*). The ΔWD mice survive postnatal starvation, maintain tissue homeostasis, and grow at the same rate as littermate controls. Importantly, for infection studies, the frequencies of B cells, T cells, and macrophages are the same as littermate controls, and ΔWD mice do not show the proinflammatory phenotype and increased interleukin-1β (IL-1β) production seen following complete loss of ATG16L1 (*33*). ΔWD mice have been used to study influenza virus infection and for production of BMDMs to study antigen presentation and responses to commensal yeasts (*22*, *34*, *35*). Furthermore, cre recombinase technology can be used to direct tissue-specific loss of the WD domain to determine the role played by LAP and CASM in different tissue types during infection (*34*).

Mice lacking Rubicon are also defective in LAP and show defects in control of *Aspergillus fumigatus* (*19*). Rubicon is a multidomain adaptor protein upstream of ATG16L1 and has multiple roles in addition to stabilizing the NADPH/NOX2 complex (*18*, *19*). These include inhibition of canonical autophagy and inhibition of endocytosis and inflammatory cytokine production following TLR signaling (*18*). Rubicon deletion has also been used in zebrafish embryo model to study LAP during *Salmonella* infection (*36*).

## AN OVERVIEW OF THE ROLE PLAYED BY LAP/CASM DURING PATHOGEN INFECTION

**Fig. 5.**
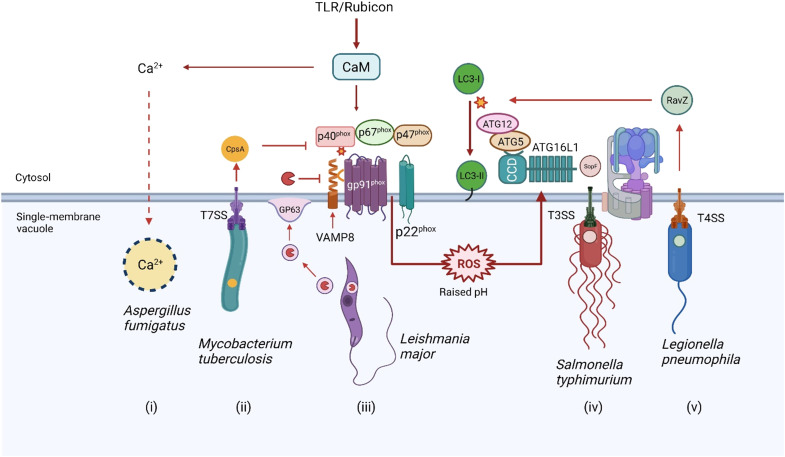
Overview of interactions between pathogens and LAP/CASM. Pathogen entry activates TLR signaling followed by Rubicon-mediated assembly of the p67^phox^:p40^phox^:p47^phox^ NADPH/NOX2 (gp91^phox^, p22phox) complex on the vacuole. Production of ROS raises vacuolar pH to activate the V-ATPase-ATG16L1 axis to conjugate LC3 to vacuolar membranes. The V-ATPase-ATG16L1 axis can also be activated when vacuolar membranes are damaged during assembly of membrane pores and translocons by pathogens. Several pathogens inhibit this pathway to slow delivery to lysosomes. Melanin in the spores of *Aspergillus* sequesters Ca^++^ required for recruitment of calmodulin (CaM) to the phagosome membrane. (**i**) CaM facilitates assembly of the p91^phox^:p40^phox^:p47^phox^ complex. *Mycobacterium* use a type 7 secretion system (T7SS) to generate a 5-nm pore to deliver CpsA into the cytosol (**ii**). CpsA binds p40^phox^, and p47^phox^ inhibits ROS production. *Leishmania* secrete exosomes containing GP63 (**iii**), which fuse with the phagosome membrane to deliver GP63 into the cytosol. GP63 is a metalloprotease that degrades VAMP to slow assembly of the p67^phox^:p40^phox^:p47^phox^ complex. *Salmonella* assemble a type 3 secretion system (TSS3) to deliver SopF into the cytosol (**iv**). SopF inhibits binding of the WD domain of ATG16L1 to V-ATPase to inhibit the V-ATPase-ATG16L1 axis and conjugation of LC3. *Legionella* assemble a type 4 secretion system (T4SS) to deliver RavZ into the cytosol (**v**). RavZ is a protease that releases LC3 from the vacuole by deconjugating LC3 from PE.

The ways that recruitment of LC3 to vacuoles by LAP/CASM can affect a variety of pathogens are summarized in Fig. 5. This is intended to provide a guide to the more detailed accounts that follow. The pathogens are presented on a background where TLR signaling has stabilized the NADPH oxidase complex through Rubicon. In most cases, it is thought that pathogens can further activate LAP/CASM when they raise vacuolar pH by generating pores in endosome or phagosome membranes. Bacteria assemble specialized secretion systems, e.g., the type 3 secretion system (T3SS) of *Salmonella*, to deliver the products of pathogenicity islands into the cytosol of the host. These translocons may generate pores in endosome membranes that can activate the V-ATPase-ATG16L1 axis to recruit LC3. At the same time, TLR signaling can lead to assembly of the NOX2 complex (p91, p40, and p47) to generate ROS, which, in turn, can increase vacuolar pH and activate the V-ATPase-ATG16L1 axis to recruit LC3. Many microbes have evolved ways to inhibit LC3 recruitment, and this generally involves inhibition of the NADPH/NOX2 complex and/or inhibition of the V-ATPase-ATG16L1 axis. Details are given in the figure legends and in the text below.

Release of pore-forming toxins and phospholipases into endosomes and phagosomes can cause further membrane damage, allowing release of Ca^2+^ into the cytosol to activate membrane repair pathways. These pathways recruit damage sensors, such as galectins that bind sugars that would not normally be exposed to the cytosol, and ubiquitin-conjugating enzymes (*37*). The sensors bind LC3 in autophagosome membranes to direct damaged membranes, and exposed pathogens to lysosomes for degradation. These damage repair pathways often run in parallel with LAP/CASM, making the interpretation of LC3 recruitment to vacuoles difficult, particularly when reading some early studies when the potential role of LAP/CASM in the control of pathogens was less well recognized. Furthermore, many pathogens make virulence factors to inhibit LAP/CASM, making it difficult to follow LAP/CASM pathways until mutant strains lacking the virulence factors become available. A general theme is, however, emerging where LAP/CASM pathways are important early during infection when pathogens are within the endolysosome system. Canonical autophagy is reserved for the removal of pathogens that escape into the cytosol.

## VIRUSES

### Influenza A virus

Respiratory viruses such as severe acute respiratory syndrome coronavirus 2 (SARS-CoV-2) [coronavirus disease 2019 (COVID-19)] and influenza A virus (IAV) cause pandemic infections where lung inflammation, cytokine storm syndrome, and pneumonia lead to high mortality. IAV infects airway and lung epithelial cells and is cleared by innate and acquired immune responses; however, excessive cytokine production during infection leads to inflammation and lung injury. Delivery of viruses to lysosomes provides an important defense against infection that can reduce virus yields and attenuate inflammatory responses. In common with many viruses, IAV activates the SNX5-ATG14 axis described above (Fig. 4, i to iv) (*32*). Infection of cells by IAV also induces redistribution of LC3 to intracellular vesicles and the plasma membrane (*38*). This redistribution is mediated by the viral M2 protein that acts as the proton channel to raise the pH of endosomes (Fig. 4, v to viii). Redistribution of LC3 by M2 requires the WD domain of ATG16L1 and the V-ATPase-ATG16L1 axis, and by implication LAP/CASM (*22*, *27*). The role played by LAP/CASM during IAV infection in vivo has been tested using ΔWD mice that lack the WD domain of ATG16L1 (*34*). Mice with systemic loss of the WD domain in all tissues were highly sensitive to infection with low-pathogenicity murine-adapted IAV, leading to extensive IAV replication in the lung, cytokine storm, and high mortality. Tissue-specific targeting of the ΔWD mutation to myeloid cells and adoptive transfer of immune cells showed that LAP/CASM in lung epithelial cells, rather immune cells and phagocytes, protected mice from lethal IAV infection. Parallel in vitro studies showed that the WD domain of ATG16L1 reduced fusion of IAV with endosome membranes and reduced cytokine production by delaying recognition of viral RNA by IFN sensors (*34*). Together, these studies establish LAP/CASM in airway epithelial (rather than professional phagocytes) as a previously unidentified innate defense that can restrict IAV infection and lethal cytokine storm.

### Picornaviruses

Infection of cells with picornaviruses induces the formation of LC3 puncta and double-membrane vesicles (DMVs) (*39*). The DMVs were first discovered during early electron microscopy of cells infected with viruses in the 1960s, and their precise origins still remain unclear (*40*). It is, however, generally believed that the DMVs provide a platform to concentrate the viral replicase proteins that generate new genomes from viral RNA templates (*41*). This increases the efficiency of replication and protects double-stranded RNA intermediates from recognition by IFN signaling pathways. The double-membrane topology of the picornavirus DMVs suggests that they might be formed by canonical autophagy pathways, and the observation that virus yields can increase 10-fold following activation of autophagy suggests that autophagy can facilitate replication (*39*, *42*). Parallel studies have, however, suggested that the membranes required for picornavirus replication may be generated by manipulation of proteins involved in trafficking membranes within the secretory pathway. The 3A nonstructural proteins of poliovirus and coxsackievirus B3 (CB3), for example, bind to Golgi membranes and activate the Arf1 guanosine triphosphatase (GTPase) (*43*–*45*). This leads to activation of phosphatidylinositol 4-kinase IIIβ (PI4-IIIβ) and increased production of PI4P that facilitates binding of the RNA-dependent RNA polymerase.

CB3 is a (+) single-stranded RNA virus of the Picornaviridae family. Inhibition of autophagy reduces CB3 replication (*46*). Systematic study of the role played by autophagy pathways in formation of DMVs showed formation of LC3 puncta, and lipidated LC3II required ATG5 and ATG16L1 but was independent of the ATG13 and FIP200 components of the ULK1 initiation complex (*47*). ULK1-independent LC3 lipidation has also been reported for poliovirus (*48*). Analysis of downstream autophagy pathways showed that LC3 lipidation was also independent of WIPI2 and ATG9A. Next, the investigators studied the role played by phosphatidylinositol 4-kinase IIIβ (PI4-IIIβ) that is recruited to the ER by the 3A nonstructural protein of CB3 and found that LC3 lipidation was markedly reduced following silencing of PI4-IIIβ expression. The precise role played by PI4-IIIβ and PI4P in virus-mediated LC3 lipidation is not clear, but it is known that the CCD domain of ATG16L1 binds PI4P directly and this may allow the virus to recruit the LC3 conjugation machinery directly to the sites of virus replication. Generation of LC3 puncta by CB3 is also reduced following knockdown of *Snx5* expression, suggesting that lipidation is facilitated by recruitment of ATG14 and the PI3K complex to endosome membranes (*32*).

### Foot and Mouth Disease Virus

Studies on the role played by autophagy during foot and mouth disease virus (FMDV) infection show induction of LC3 puncta and LC3 lipidation very early during infection. This occurs at a time when capsids could be seen colocalized with LC3 in endocytic compartments, suggesting activation of LC3 lipidation during cell entry (*49*). Furthermore, the LC3 puncta could be induced by ultraviolet-inactivated virus and by empty capsids independently of replication. These results suggest that entry of FMDV into cells triggers recruitment of LC3 to single-membrane endosomes, but a systematic analysis of the role played by the ULK1 complex or the WD domain of ATG16L1 has not been reported, so it is not possible to rule out activation of autophagy. It will be interesting to determine whether FMDV induces binding of SNX5 to ATG14 to induce LC3 lipidation of endosomes, and whether this is activated by the integrins used for FMDV cell entry.

## BACTERIA

### Salmonella

*Salmonella* serovars are foodborne pathogens that cause a range of gastrointestinal diseases. Oral ingestion of contaminated water or food results in delivery of bacteria to the small intestine where they enter gastrointestinal epithelial cells by endocytosis. Key stages in bacterial entry rely on T3SS that inject effector proteins encoded by pathogenicity islands across host cell membranes. Effectors encoded by pathogenicity island 1 (SPI-1) are injected across the plasma membrane and stimulate actin rearrangements to facilitate invasion of the cell. *Salmonella* enter endosomes that recruit markers for early and late endosomes (Rab5, Rab7, and Rab9) and lysosomes (LAMP1). The endosomes also recruit V-ATPase, which lowers the pH in the lumen of the endosome, and this activates SPI-2. The effectors encoded by SPI-2 are injected into the cytosol and modify the endosome to produce *Salmonella*-containing vacuoles (SCVs), which are thought to protect the bacteria from recognition by cytosolic sensors of infection and degradation in lysosomes through autophagy. The SPI-2 effector SifA, for example, interacts with endosome trafficking proteins to protect salmonella by slowing delivery of lysosomal enzymes to the SCV, and SifA is required for the formation of membrane tubules that extend from the SCV along microtubules. These *Salmonell*a-induced tubules may connect with endocytic pathways to bring nutrients into the SCV (*50*).

**Fig. 6.**
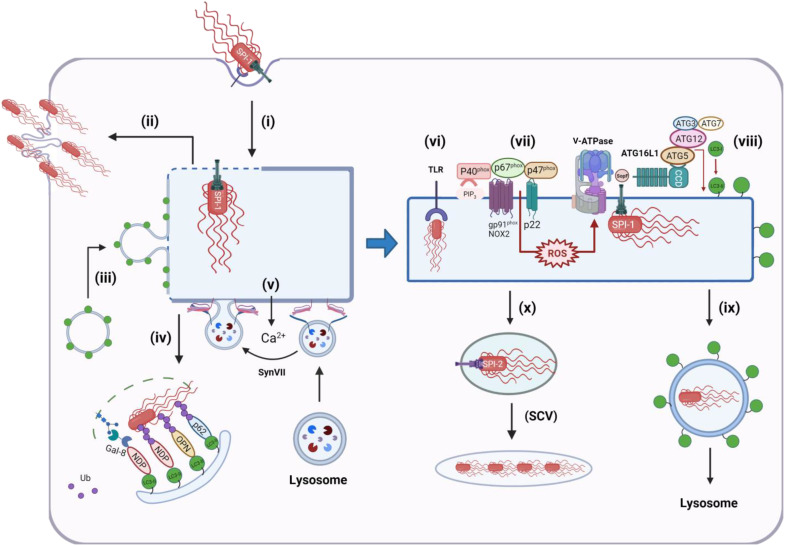
Recruitment of LC3 to membranes during *Salmonella* infection. Effectors encoded by SPI-1 are injected across the plasma membrane and stimulate actin rearrangements to facilitate invasion of the cell (**i**). A small number (10 to 20%) of bacteria escape to the cytosol and can hyperreplicate, causing cell death (**ii**). Assembly of the T3SS translocon generates pores in the endosome that can induce recruitment of LC3 and autophagosomes (**iii**) to stabilize the endosome and slow release of *Salmonella* into the cytosol, and at the same time, damaged endosomes and exposed bacteria can be ubiquitinated and recognized by cytosolic autophagy cargo sensors such as galectin 8 (Gal-8), NDP52, optineurin (OPN), and p62 and delivered to lysosomes for degradation (**iv**). Release of Ca^2+^ into the cytosol can activate synaptotagmin VII (SynVII)–mediated lysosome repair (**v**). *Salmonella* retained in early endosomes trigger TLR signaling (**vi**) to activate NADPH oxidase to generate ROS, leading to assembly of V-ATPase (**vii**). Pores generated by the T3SS translocon can also induce recruitment of V-ATPase to vacuoles containing *Salmonella*. V-ATPase binds the WD domain of ATG16L1, leading to recruitment of the LC3 conjugation complex (ATG5-ATG12, ATG3, and ATG7) and conjugation of LC3 to PE in the vacuole membrane (**viii**) and transport to lysosomes (**ix**). Binding of the WD domain to V-ATPase is inhibited by virulence factor SopF. If vacuoles containing salmonella are repaired, they maintain low pH, allowing effectors encoded by SPI-2 to modify the endosome to produce salmonella-containing vacuoles (SCVs), which provide a niche for slow replication in membrane compartments separated from autophagy and other innate sensors of infection (**x**).

*Salmonella* infection triggers the formation of LC3 puncta that results from the formation of autophagosomes and from the translocation of LC3 to single-membrane endolysosome compartments. Early on during infection, a small number (10 to 20%) of *Salmonella* escape into the cytosol. This occurs as early as 15 min after invasion and continues for an hour (Fig. 6, i). In epithelial cells, the delivery of *Salmonella* to nutrient-rich cytosol leads to hyperreplication (>100 bacteria per cell) and host cell death (Fig. 6, ii). Hyperreplication does not occur in fibroblasts or macrophages. Damage to endosomes during *Salmonella* entry and escape has the potential to recruit LC3 onto membranes via autophagy, and this may stabilize the endosome and slow release of bacteria into the cytosol (Fig. 6, iii). Endosomes damaged by *Salmonella* can, for example, be ubiquitinated and recruit p62 and LC3 (*51*–*54*). Studies of endosomes containing latex beads coated with cationic polymers used as surrogate bacteria (*51*) showed ubiquitin-dependent recruitment of ULK1, WIPI2, and ATG14L, suggesting canonical autophagy; however, part of the mechanism involves direct binding of ubiquitinated proteins to the WD domain of ATG16L1, rather than binding of WIPI2 to PIP_3_. Later work using an RNA interference (RNAi) screen showed that early membrane repair during *Salmonella* entry requires autophagy proteins such as ATG5-ATG12 and ATG16L1 required for LC3 conjugation (*55*). LC3 recruitment to Rab5-positive structures required activation of SPI-1 (T1) and occurred early at 20 min and peaked at 40 to 60 min and dissociated following induction of SPI-2. Together with the observation that SCV maturation was slowed in the absence of ATG5, it is likely that autophagy-dependent membrane repair stabilizes the endosome and slows release of *Salmonella* into the cytosol. This, in turn, favors progression to the SCV and expression of SPI-2 (T2) effectors. Interestingly, silencing of FIP200 required for canonical autophagy did not affect membrane repair (*55*), suggesting that LC3 recruitment to the SCV may be through LAP/CASM; however, a requirement for ATG9 and ULK1 argues against this.

Much work has focused on the role played by canonical autophagy in the control of *Salmonella* that enter the cytosol from damaged endosomes. Lipopolysaccharides exposed on the surface of cytosolic *Salmonella* are ubiquitinated by the E3 ubiquitin ligase RNF213 (Fig. 6, iv), leading to recruitment of autophagy cargo receptors p62/SQSTM1, optineurin, and NDP52, which bind to LC3 in autophagosome membranes (*56*). Vacuolar damage caused by *Salmonella* is recognized by a cytosolic lectin called galectin-8 (*37*) that facilitates autophagy by recruiting NDP52 to vacuole remnants associated with bacteria (Fig. 6, iv). Silencing *RNF213*, Atg5, Atg7, Atg16L1, or galectin or pharmacological inhibition of autophagy increases *Salmonella* replication, suggesting that autophagy and degradation in lysosomes protect cells from infection (*37*, *53*, *54*, *56*).

For *Salmonella* that are retained in endosome assembly of the T3SS protein, translocation machinery generates pores between 2 and 5 nm diameter that can alter the balance of ions across the SCV and endosome. The entry of Ca^2+^ into the cytosol triggers lysosome-mediated membrane repair where lysosomes fuse with membranes to seal the pore (Fig. 6, v). Synaptotagmin VII senses Ca^2+^ entering cells through the T3SS translocon and triggers fusion of lysosomes with endosomes and SCV containing *Salmonella* (*57*). As with recruitment of LC3 and autophagosomes (*55*), lysosome fusion is thought to stabilize the SCV and protect cells from infection by slowing entry *of Salmonella* into the cytosol.

In epithelial cell lines, LC3 is directed to vacuoles containing *Salmonella* by a pathway dependent on ROS, again suggesting involvement of LAP/CASM (*4*). As with autophagy-dependent membrane repair, LC3 recruitment is seen within 60 min of cell entry, suggesting translocation of LC3 to endosomes damaged by SPI-1 before expression of SPI-2 effectors. Epithelial cells do not express NOX2 but do express p22phox. LC3 translocation was inhibited by inhibition of NADPH oxidase activity by DPI and siRNA for *p22phox*. Silencing *p22phox* resulted in increased replication at 6 to 8 hours, consistent with LC3 slowing escape of *Salmonella* into the cytosol from damaged endosomes. This raises the possibility that LC3 is recruited to endosomes containing *Salmonella* by a LAP/CASM pathway activated by TLR signaling. Recruitment of LC3 to *Salmonella* has been demonstrated in cells lacking ATG9, a membrane protein that is essential for autophagosome formation (*58*). Live cell imaging of control Atg9^+/+^ cells showed recruitment of the PI3K complex (ATG4), WIPI-1, ATG5, and LC3 to *Salmonella* in the cytosol, consistent with a role for canonical autophagy in engulfment of *Salmonella* by autophagosomes. Interestingly, analysis of Atg9^−/−^ MEFs showed that ATG5, ATG16L1, and green fluorescent protein (GFP)–LC3 could be recruited to *Salmonella* independently of ATG9 and the PI3K. Correlative light electron microscopy showed *Salmonella* surrounded by a single membrane in Atg9^−/−^ cells, and in cells lacking the FIP200 protein also required for canonical autophagy. LC3 became associated with *Salmonella* contained within a membrane vacuole within 10 min of infection, consistent with recruitment of LC3 to endosomes and/or SCV damaged by SP1 effectors, rather than by cytosolic bacteria. Together, these results again show that LC3 can be recruited to single membranes surrounding *Salmonella* early during infection by pathways independent of canonical autophagy.

### Sop-F Inhibits the V-ATPase-ATG16L1 axis

Quantitative secretome profiling by liquid chromatography–tandem mass spectrometry (LC-MS/MS) identified SopF (STM1239) as SPI-1 effector protein secreted into the cytoplasm by *Salmonella* through T3SS (*59*). SopF facilitates replication in macrophages and virulence in mice. SopF binds phosphoinositides and locates to membranes in cells where it concentrates in ruffles during bacterial entry, and around intracellular bacteria (*60*). Δ*sopF Salmonella* show increased exposure of glycans recognized by galectin-8 and increased colocalization with p62 and LC3 early during infection (1 hour), suggesting that SopF stabilizes the SCV. Parallel work using an unbiased genetic screen identified SopF as an effector protein able to inhibit recruitment of LC3 to SCVs (*26*). Inhibition of LC3 recruitment to vacuoles was independent of bacterial species, and ectopic expression of SopF inhibited recruitment of LC3 to endosomes damaged by polystyrene beads.

Δ*sopF Salmonella* were used in a CRISPR-Cas9 screen to identify host factors required for LC3 translocation to vacuoles (*26*). Interestingly, LC3 recruitment did not require FIP200, which is essential for canonical autophagy, but did require the LC3 conjugation machinery (ATG3, ATG5:ATG12, ATG7, and ATG16L1). Incubation of cells with impermeable dyes showed that LC3 recruitment was coincident with release of the dye from vacuoles and was dependent on T3SS. As with a previous study (*55*), this suggested that LC3 recruitment is triggered by vacuolar damage induced by assembly of T3SS. Further work showed that vacuolar damage induced binding of ATG16L1 to V-ATPase in the membrane of the SCV (Fig. 6, v and vi) (*26*). This provided a pathway for recruiting LC3 conjugation machinery to the cytoplasmic face of vacuoles containing bacteria. Importantly, binding of ATG16L1 to V-ATPase required the WD domain of ATG16L1 (*26*), again indicating parallels with recruitment of LC3 to membranes by LAP/CASM.

The crystal structure of Sop-F (*61*) revealed close similarity with adenosine diphosphate (ADP)–ribosyl transferases, which add ADP-ribose to proteins. ADP-ribose pull-down experiments demonstrated that Sop-F transferred ADP-ribose to Gln^124^ in the ATP6VOC subunit of V-ATPase, and that this blocked binding of the WD domain of ATG16L1 (*26*). Mechanistically, it is thought that membrane pores resulting from assembly of the T3SS translocon induce a conformational change in V-ATPase and/or raise vacuolar pH allowing V-ATPase to bind the WD domain of ATG16L. This, in turn, recruits the LC3 conjugation machinery to the vacuole independently of the ULK1 and PI3K complexes required for canonical autophagy. A product of T3SS encoded by *Burkholderia pseudomallei* also inhibits LAP to slow killing in phagolysosomes (*62*). It will be interesting to see whether this involves inhibition of the V-ATPase-ATG16L1 axis.

### Salmonella in vivo

Murine infection models show that *Salmonella* induce the formation of LC3 puncta in intestinal epithelial cells (*63*, *64*). LC3 puncta were dependent on the innate immune adaptor protein MyD88 that is downstream of many TLRs that recognize Pathogen-associated molecular patterns (PAMPs) expressed by bacteria (*64*). The observation that dissemination of *Salmonella* and enteric bacteria to spleen and liver increases following cre-lox–mediated loss of Atg5 or Atg16L1 from intestinal epithelial cells raises the possibility that recognition of *Salmonella* by TLRs may activate autophagy pathways to protect against infection in vivo (*63*, *64*). The detection of double-membrane vacuoles surrounding *Salmonella*, rather than single membranes, suggests autophagy; however, these electron micrographs may represent the removal of *Salmonella* by autophagy late during the infectious cycle, rather than early recruitment of LC3 triggered by LAP/CASM (*64*).

The role played by LAP/CASM during *Salmonella* infection in vivo has been tested directly using zebrafish embryos (*36*). Live cell imaging of embryos expressing GFP-LC3 showed recruitment of LC3 to vacuoles containing *Salmonella* expressing mCherry. The vacuoles were formed predominantly in macrophages and neutrophils and, as judged by electron microscopy, were surrounded by single rather than double membranes. A morpholino knockdown demonstrated that embryo survival, and recruitment of LC3 to vacuoles following infection, required ATG5 but did not require ATG13, a component of the ULK1 complex required to initiate canonical autophagy. Interestingly, knockdown of Rubicon or the p22 subunit of NADPH oxidase reduced LC3 recruitment to vacuoles and increased susceptibility of embryos to infection. Together, the results show that LAP in phagocytic cells plays an important role in protection against *Salmonella* infection in vivo.

### Listeria

*Listeria* are Gram-positive bacteria that can cause severe foodborne infections, particularly in immunocompromised individuals. *Listeria* are primarily controlled during phagocytosis by macrophages, allowing bacteria to be killed following delivery of lysosomal enzymes to phagosomes. *Listeria* can escape from the phagosome by generating the pore-forming toxin listeriolysin O (LLO) and two phospholipase enzymes PI-PLC and PC-PLC. *Listeria* entering the nutrient-rich cytosol replicate rapidly and then use ActA-mediated polymerization of actin to generate actin “rockets” at one end of the bacteria that propel them into neighboring cells.

**Fig. 7.**
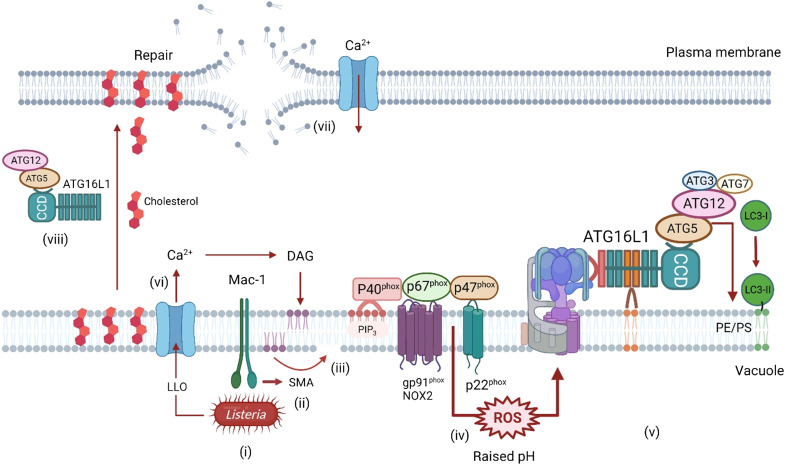
Recruitment of LC3 to membranes during *Listeria* infection. Binding of *Listeria* to Mac-1 in vacuoles in macrophages (**i**) activates sphingomyelinase (SMA), which removes the phosphocholine head group from sphingomyelin to generate ceramide (ii and **iii**). Ceramide-enriched microdomains facilitate assembly of the NADPH oxidase/NOX2 complex and enhance production of ROS (**iv**), which leads to assembly of V-ATPase and recruitment of ATG16L1:ATG5-ATG12 to conjugate LC3 to the vacuole membrane (**v**). LLO is a pore-forming toxin release by *Listeria*. Damage to the vacuole and subsequent entry of Ca^2+^ into the cytosol (**vi**) induce production of DAG, which activates the NADPH oxidase/NOX2 complex directly. Damage to the plasma membrane by LLO and entry of Ca^2+^ into the cytosol trigger a membrane repair pathway, which is dependent on the WD domain of ATG16L1 and ATG5-ATG12 (**vii** and **viii**). This may involve transport of cholesterol from vacuoles to the plasma membrane and lysosome-mediated membrane repair.

In fruit flies, recognition of *Listeria* cell wall peptidoglycan by pattern recognition receptor PGRP-LE leads to Atg5-dependent formation of LC3-positive autophagosomes, and intracellular bacteria are held within double-membrane vacuoles, suggesting capture by canonical autophagy (*65*). Atg5 silencing increases intracellular growth of *Listeria* and decreases survival of flies, showing that in flies autophagy plays a protective role during *Listeria* infection. Similar experiments show increased sensitivity to *Listeria* infection when mice lack *atg5* in myeloid cells, again suggesting a protective role for autophagy. In vitro studies using RAW264.7 macrophages show peak association of LC3 with intracellular bacteria 1 hour after cell entry. Close examination of phagosomes showed that vacuoles containing *Listeria* lacked ubiquitin and p62 and have a single membrane suggesting capture by LAP/CASM (*66*, *67*). LC3 association is lost once bacteria enter the cytosol where they become targets for canonical autophagy. LC3 recruitment may be prevented by ActA, which inhibits host E3 ligases to reduce subsequent recruitment of ubiquitin and autophagy adaptor proteins p62 and NDP52. These results suggest that, in mammalian cells, LC3 recruitment to *Listeria* can be directed by autophagy and LAP/CASM, but as seen for *Salmonella*, LAP/CASM is the predominant pathway for recruitment to phagosomes early during entry while canonical autophagy targets *Listeria* in the cytosol (Fig. 7). LC3 recruitment to vacuoles early during infection is independent of ULK1, requires expression of pore-forming toxin LLO, and can lead to the formation of large vacuoles called SLAPs (spacious *Listeria* containing phagosomes) (*66*, *67*). LC3 recruitment to vacuoles and SLAP maturation were greatly reduced when NADPH oxidase was inhibited by DPI and was reduced in BMDMs lacking NADPH oxidase activity (*67*, *68*). LC3 recruitment early during infection also required generation of diacylglycerol (DAG) by host phospholipase D (PLD) and phosphatidic acid phosphatase (*66*). Studies using *cybb^−/−^* BMDM lacking the NOX2 required for ROS production have revealed a ROS-independent pathway for recruitment of LC3 to vacuoles damaged by LLO or pore-forming toxins from *S. aureus* (*69*). This has been called PINCA for pore-forming toxin–induced noncanonical autophagy. Interestingly, LC3 conjugation in response to PINCA increases fusion of phagosomes with lysosomes but does not make a major contribution to killing of *Listeria*. This could be because of reduced ROS production.

Killing of *Listeria* by tissue-resident macrophages requires expression of Mac-1 (Fig. 7) (*67*). Mac-1 is an amb2 integrin composed of CD11b and CD18 that can bind extracellular matrix and complement (*70*). Peritoneal macrophages from mice deficient in the CD11b subunit of Mac-1 produce low levels of ROS when incubated with *Listeria* and show reduced recruitment of LC3 to phagosomes containing *Listeria* (*67*). Further work shows that recognition of *Listeria* by Mac-1, rather than other pattern recognition receptors such as MyD88-dependent TLRs or NOD2, was required for activation of NOX2 and ROS production. Mac-1 induces ROS by activating acid sphingomyelinase, which generates ceramide in the phagosome membrane to recruit NOX2 and also to promote fusion with lysosomes. Mice with loss of ATG7 from myeloid cells showed increased bacterial burden, indicating that activation of CASM/LAP by Mac-1 is important for elimination of *Listeria* in vivo.

LLO is a pore-forming toxin that enhances growth of *Listeria* in vacuoles and allows the bacteria to escape into the cytosol. Similarly, pneumolysin toxin from *Streptococcus pneumoniae* generates pores by binding cholesterol in membranes and can activate CASM/LAP (*71*). As with *Salmonella,* the flux of Ca^2+^ into the cell induced by the toxin stimulates membrane repair pathways where lysosomes fuse with the plasma membrane or damaged endosomes. Recent work shows that this process requires autophagy protein ATG16L1 (*72*), and interestingly, membrane repair required the WD domain of ATG16L1 and was compromised in cells expressing ATG16L1 carrying the T300A risk allele for Crohn’s disease. At first glance, this would suggest involvement of LC3 recruitment via CASM/LAP, but repair did not require Rubicon and did not result in recruitment of LC3 or ATG16L1 to sites of repair. Instead, the WD domain of ATG16L1 appears to be required for maintaining the correct distribution of cholesterol in cells, and this may be important for lysosome exocytosis during membrane repair. This noncanonical role for ATG16L1 inhibits damage inflicted by LLO and slows cell to cell spread of *Listeria monocytogenes* (*72*).

### Legionella

*Legionella* are Gram-negative bacteria that are parasites of phagocytic protozoa such as amoebae, but they can infect humans if they get access to the respiratory tract where they infect alveolar macrophages leading to a pneumonia called Legionnaire’s disease. Survival in phagocytes is dependent on a type 4 secretion system (T4SS) (Dot/Icm) that assembles over 20 different proteins into a pore able to span the inner and outer envelopes of the bacteria allowing secretion of virulence factors into the cell. *Legionella* entering alveolar macrophages by phagocytosis are surrounded by a membrane compartment called the *Legionella*-containing vacuole (LCV). The vacuole protects the bacteria from the phagosome-lysosome system by recruiting small GTPases such as Rab1 and Arf1, which recruit ER- and Golgi-derived membrane vesicles to deliver ER proteins to the vacuole. The LCV also acquires autophagy markers ATG7 and LC3 possibly as a result of pore formation by type IV virulence factors (*73*). *Legionella* are, however, able to inhibit autophagy and degradation in lysosomes by secreting the RavZ protein (Fig. 5, v), which acts an LC3 deconjugation enzyme to remove LC3 from autophagosome membranes (*74*). Interestingly, studies of the *Legionella dumoffi* strain, which does not encode RavZ, show recruitment of LC3 to vacuoles containing bacteria by LAP in macrophage cell lines (*75*). Unlike the *Legionella pneumophila* strain, the LCVs formed around *L. dumoffi* had single membranes and were negative for ubiquitin, galectin-8, NDP52, and p62. In common with LAP, recruitment of LC3 was independent of ULK1 but did require NADPH oxidases and DAG and was facilitated by TLR signaling and Rubicon. Live cell imaging showed that LC3 recruitment to LCVs was followed by acidification and degradation of bacteria, indicating that LAP provides a defense against infection that may be subverted by deconjugation of LC3 by the RavZ protein of *L. pneumophila* (*74*).

### Mycobacterium tuberculosis

It has been known for many years that *Mycobacterium tuberculosis* (*M.tb*) survives in macrophages by evading degradation in lysosomes (*76*). The mycobacterium activates pathogen recognition receptors, but very few phagosomes recruit LC3, suggesting that wild-type *M.tb* can inhibit CASM/LAP. A yeast two-hybrid screen using the reading frames of proteins predicted to be secreted by *M.tb* identified CpsA as a protein able to bind autophagy cargo protein NDP52 and might therefore modulate autophagy (*77*). CpsA is secreted by type VII secretion (ESX-1 translocon) that creates a 5-nm pore in the inner membrane of the bacteria, allowing virulence factors to leave the bacteria through the cell wall and the outer hydrophobic mycomembrane. Simultaneous secretion of membrane destabilizing factors is thought to damage the phagosome, allowing delivery of CpsA into the cytosol of the macrophage (Fig. 5, ii). At the same time, release of DNA, either from *M.tb* or mitochondria responding to infection, can activate the cGAS/STING pathway, leading to type 1 IFN production and noncanonical recruitment of LC3 to endolysosome membranes via the V-ATPase-Atg16L1 axis (*78*). Interestingly, *M.tb* strains lacking CpsA (Δ*cpsA*) show increased recruitment of LC3 to phagosomes containing bacteria and increased delivery of *M.tb* to LAMP1-positive compartments for degradation. The degradation of mutant *M.tb* did not require ULK1 or ATG14 or cGAS but was dependent on Rubicon and NADPH oxidase, showing that CASM/LAP was responsible for clearing the Δ*cpsA* mutant. Further analysis showed that exogenous expression of CpsA in macrophages inhibited recruitment of p47^phox^ and p40^phox^ to phagosomes containing zymosan or wild-type *M.tb*. Crucial in vivo experiments showed that Δ*cpsA* strains failed to establish infections in mice unless mice carried mutations in NOX2, demonstrating that CpsA protects *M.tb* from CASM/LAP in vivo by inactivating NADPH oxidase (*77*).

### Shigella flexneri

*Shigella flexneri* are Gram-negative bacteria causing bacterial dysentery and are one of the leading causes of deaths of infants in developing countries. *Shigella* reaching the colon and rectum translocate across epithelial barriers where they are ingested by macrophages, leading to rapid replication and cell death. *Shigella* released from macrophages enter epithelial cells by phagocytosis/endocytosis where expression of T3SS damages the vacuole, allowing escape into the cytosol. Expression of outer membrane protein VirG (IcsA) seeds actin assembly at one pole of the bacteria through interactions with N-WASP and the Arp2/3 actin polymerization complex to propel the bacteria into neighboring cells. At the same time, VirG binds ATG5 and this may initiate capture of cytosolic *Shigella* by canonical autophagy. This process is inhibited by the IcsB protein that is secreted into the cytosol via T3SS to compete with ATG5 for binding to VirG (*79*).

More recent work has shown recruitment of LC3 to *Shigella* during cell-to-cell spread. *Shigella* are carried into neighboring cells within membrane protrusions generated by actin polymerization. The protrusions are engulfed by the endolysosome system of the recipient cells where acidification switches on T3SS, leading to membrane damage that triggers recruitment of LC3, possibly by LAP/CASM. At the same time, expression of IcsB and VirA is thought to destabilize the new vacuole to allow escape of *Shigella* into the cytosol of the recipient cell, before they are killed by fusion with lysosomes (*80*).

## FUNGI AND YEASTS

### A. fumigatus

*A. fumigatus* is a major airborne fungal pathogen where predisposition to infection is increased by steroid treatment, by chemotherapy, or through mutations to NADPH oxidase as seen in chronic granulomatous disease. *Aspergillus* is transmitted following inhalation of dormant conidia (spores) into the airways where they are controlled through phagocytosis by alveolar macrophages and subsequent activation of proinflammatory responses. The cell walls of dormant conidia are covered by hydrophobic rodlet proteins that conceal PAMPs. The rodlets are held in the cell wall by a glycosylphosphatidylinositol anchor that is degraded during germination, allowing the subsequent exposure of PAMPs to activate strong innate immune responses.

Exposed β-glucan is recognized by Dectin-1, and Syk kinase/NADPH signaling activates recruitment of LC3 to phagosomes by LAP (*81*). Detailed studies have shown that conidia unable to express rodlet proteins (Δ*rod*) expose β-glycan but do not activate LAP/CASM unless the conidia carry a second mutation, Δ*pksp*, that prevents expression of cell wall melanin (*82*). The combined loss of rodlets and melanin from conidia increased killing in lysosomes and reduced virulence in immunocompromised mice, suggesting that melanin prevents innate immune recognition and elimination of conidia by LAP/CASM. A comparison of cell signaling pathways induced by mutant and wild-type conidia showed that cell wall melanin greatly reduced production of ROS following Dectin-1/Syk signaling from macrophage phagosomes by preventing assembly of p22phox into the NADPH-oxidase complex. The loss of ROS impairs subsequent recruitment of LC3 via LAP/CASM. The topological problem of how melanin inside the phagosome would affect assembly of the NADPH oxidase complex on the cytosolic side of the vacuole was solved by the discovery that, in Δ*rod* conidia lacking melanin, calmodulin (CaM) is recruited from the cytosol to phagosomes following activation of LAP/CASM (*83*). Recruitment of LC3 to phagosomes and killing of *Aspergillus* were inhibited by inhibitors of CaM or calcium/CaM-dependent kinase II (CAMKII). A comparison of Ca^2+^ release from phagosomes showed that phagosomes containing wild-type strains released less of Ca^2+^ than melanin^−/−^ strains. This suggested that melanin sequestered Ca^2+^ within the phagosome, blocked recruitment of CaM, and reduced Ca^2+^-CaM signaling. Remarkably, a single-nucleotide polymorphism (CALM1 CC) that reduces the activity of the CaM promoter has been found in patients at risk of *Aspergillus* infection, suggesting a role for impaired Ca^2+^-CaM signaling in preventing human fungal infection (*83*).

LAP has also been shown to target yeast (*84*). Invasive candidiasis caused by *Candida albicans* is a leading cause of hospitalization and death in immunocompromised patients. In common with *A. fumigatus*, *C. albicans* is controlled by phagocytic cells, and activation of Dectin-1 by exposed β-1,3 glucan recruits LC3 to the phagosome membrane (*84*). Phosphorylation of Dectin by Syk leads to activation of the NADPH oxidase on the phagosome and recruitment of LC3 via LAP/CASM. LAP/CASM pathways also respond to recognition of α-mannan in the cell walls of commensal yeast such as *Saccharomyces cerevisiae* and *Kazachstania unispora* in the gut microbiota where they facilitate Dectin-2 signaling and proinflammatory responses (*35*).

## UNICELLULAR PARASITES

### Leishmania

*Leishmania* are unicellular eukaryotes responsible for leishmaniasis. Promastigotes use flagella to facilitate entry into phagocytic cells and transform into a nonmotile amastigote form that can replicate by cell division. Promastigotes internalized into phagocytes use a zinc metalloprotease called GP63 to inhibit phagolysosome fusion and prevent degradation in lysosomes. GP63 is secreted from the *Leishmania* in exosomes that fuse with the limiting membrane of the vacuole and deliver GP63 into the cytosol (Fig. 5, iii). Recent studies using mutants lacking GP63 show that GP63 prevents recruitment of LC3 to phagosomes containing *Leishmania* during early stages of cell entry (*85*). Earlier work had shown that vesicle-associated membrane protein 8 (VAMP8) controlled recruitment of the NOX2 complex on phagosomes and that VAMP8 was cleaved by GP63 (*86*). This allows GP63 to prevent LAP/CASM by preventing production of ROS and consequent rise in vacuolar pH.

### Plasmodium

*Plasmodium* species are unicellular eukaryotic parasites that are transferred between hosts by insects such as mosquitoes. *Plasmodium vivax* infects humans where it causes malaria. It is not as lethal as *Plasmodium falciparum* but has widespread global distribution and can live in the liver in a dormant hypnozoite stage for many years. It has been known for a long time that IFN-γ can prevent liver stage infection (*87*). IFN-γ also inhibits the intrahepatocytic development of malaria parasites in vitro, but the mechanisms were unclear (*87*). IFN-γ induces LC3 lipidation and formation of LC3 puncta, suggesting a role for LC3 in clearance of *Plasmodium* (*88*). IFN-γ induces the formation of relatively small numbers of LC3 puncta per cell (between 1 and 4) that require expression of Beclin1 and ATG5 (*89*). Cells lacking Beclin1 and ATG5 were less able to eliminate parasites in response to IFN-γ, but elimination was unaffected following loss of ULK1, implicating LAP/CASM (*89*). *Plasmodium* do not enter cells by phagocytosis but use an active parasite-driven pathway that leads to the formation of a parasitophorous vacuole in the cytosol (*90*). IFN-γ does, however, increase recruitment of LC3 to parasitophorous vacuoles, and this may be through LAP/CASM. This is supported by studies of *Plasmodium berghei* where LC3 is recruited to parasitophorous vacuoles formed in hepatocytes within 20 min of cell entry. Recruitment of LC3 required ATG5 but was independent of FIP200 (*88*).

## CONCLUSIONS

The identification of pathways such as LAP and CASM, which conjugate LC3 to endosomes and phagosomes containing pathogens as they enter cells, has given a new perspective on how autophagy proteins can control infection. It is known that LC3 recruitment during infection is triggered by TLR signaling through Rubicon and ROS production and can be mediated by the V-ATPase-ATG16L1 axis, which senses a rise in vacuolar pH. It is now appreciated that pathogens can further activate LC3 recruitment when they raise vacuolar pH following membrane damage or generation of pores in endosomes or phagosomes before they gain access to the cytosol. This information will underpin the search for new pathogen-associated signals able to activate these alternative pathways of LC3 conjugation, and systematic studies of the pathways involved. There will also be a search for unknown virulence factors generated by pathogens to slow LC3 recruitment by inhibition of defined pathways such as ROS production and/or the V-ATPase-ATG16L1 axis, or through inhibition of previously unidentified pathways. Animal models that are defective in recruiting LC3 to membranes through LAP/CASM will be valuable for determining the importance of LAP and CASM in controlling infection in vivo. Key questions center on the role played by LAP and CASM at epithelial surfaces and within phagocytic cells and leukocytes, and how these influence innate and acquired immune responses.
